# Emulating a target trial of early compared with late initiation of appropriate antibiotic therapy for hospital-acquired monobacterial Gram-negative bloodstream infections

**DOI:** 10.1093/jac/dkag161

**Published:** 2026-05-13

**Authors:** Abdullah T Aslan, Yukiko Ezure, Elif Seren Tanriverdi, Osman Dağ, Cansu Cimen, Ayşe Kaya Kalem, Bircan Kayaaslan, Sevil Alkan, Bahadır Köylü, Emine Büşra Ata, Bilge Çağlar, Neşe Saltoğlu, Uğur Önal, Seçil Deniz, Onur Ural, Murtaza Öz, Mehmet Bakir, Mesut Yilmaz, Rümeysa Çakmak, Ayşe Batirel, Özlem Akdoğan, Nurcan Baykam, Çiğdem Erol, Tuğba Yanik-Yalçin, Oya Özlem Eren-Kutsoylu, Zeynep Türe-Yüce, Gamze Kalin-Ünüvar, Zuhal Özer-Şimşek, Seda Güzeldağ, Adem Köse, Mustafa Cihangiroğlu, Dilek Yağci-Caglayik, Muhammed Burak Sevinç, Zerrin Aktaş, Oral Öncül, Gülden Ersöz, Ayşe Sesin Kocagöz, Gülşen Hazirolan, Tuğçe Ünalan-Altintop, Bedia Dinç, Nazmiye Ülkü Tüzemen, Alper Akçali, Salih Maçin, Ahmet Çalişkan, Mürşit Hasbek, Cem Ergon, Yasemin Ay-Altintop, Müge Şimşek, Barış Otlu, Kay A Ramsay, Patrick N A Harris, Murat Akova, David L Paterson, the Study Group for Carbapenem Resistance (SCARE), Abdullah T Aslan, Elif Seren Tanriverdi, Osman Dağ, Cansu Cimen, Ayşe Kaya Kalem, Bircan Kayaaslan, Sevil Alkan, Neşe Saltoğlu, Uğur Önal, Seçil Deniz, Onur Ural, Murtaza Öz, Mehmet Bakır, Mesut Yılmaz, Rümeysa Çakmak, Ayşe Batırel, Özlem Akdoğan, Nurcan Baykam, Çiğdem Erol, Tuğba Yanık-Yalçın, Oya Özlem Eren-Kutsoylu, Zeynep Türe-Yüce, Gamze Kalın-Ünüvar, Zuhal Özer-Şimşek, Seda Güzeldağ, Adem Köse, Mustafa Cihangiroğlu, Dilek Yağcı-Caglayik, Muhammed Burak Sevinç, Gülden Ersöz, Ayşe Sesin Kocagöz, Gülşen Hazırolan, Tuğce Ünalan-Altıntop, Nazmiye Ülkü Tüzemen, Alper Akçalı, Salih Maçin, Ahmet Çalışkan, Mürşit Hasbek, Barış Otlu, Murat Akova, İlke Toker Önder, Seda Nihal Yücesoy

**Affiliations:** Faculty of Health, Medicine and Behavioural Sciences, UQ Centre for Clinical Research, University of Queensland, Brisbane, QLD, Australia; Sunshine Coast University Hospital, Department of General Medicine, Sunshine Coast, QLD, Australia; Faculty of Health, Medicine and Behavioural Sciences, UQ Centre for Clinical Research, University of Queensland, Brisbane, QLD, Australia; Faculty of Medicine, Department of Medical Microbiology, Inönü University, Malatya, Türkiye; Faculty of Medicine, Department of Biostatistics, Hacettepe University, Ankara, Türkiye; Department of General Internal Medicine, Infectious Diseases and Tropical Medicine, Antwerp University Hospital, Antwerp, Belgium; Ankara Yildirim Beyazit University, Faculty of Medicine, Ankara Bilkent City Hospital, Department of Infectious Diseases and Clinical Microbiology, Ankara, Türkiye; Ankara Yildirim Beyazit University, Faculty of Medicine, Ankara Bilkent City Hospital, Department of Infectious Diseases and Clinical Microbiology, Ankara, Türkiye; Faculty of Medicine, Department of Infectious Diseases and Clinical Microbiology, Çanakkale Onsekiz Mart University, Çanakkale, Türkiye; School of Medicine, Department of Internal Medicine, Hacettepe University, Ankara, Türkiye; School of Medicine, Department of Internal Medicine, Hacettepe University, Ankara, Türkiye; Cerrahpaşa Medical Faculty, Department of Infectious Diseases and Clinical Microbiology, Istanbul University, Istanbul, Türkiye; Cerrahpaşa Medical Faculty, Department of Infectious Diseases and Clinical Microbiology, Istanbul University, Istanbul, Türkiye; Faculty of Medicine, Department of Infectious Diseases and Clinical Microbiology, Uludağ University, Bursa, Türkiye; School of Medicine, Department of Infectious Diseases and Clinical Microbiology, Pamukkale University, Denizli, Türkiye; Faculty of Medicine, Department of Infectious Diseases and Clinical Microbiology, Selçuk University, Konya, Türkiye; Faculty of Medicine, Department of Infectious Diseases and Clinical Microbiology, Sivas Cumhuriyet University, Sivas, Türkiye; Faculty of Medicine, Department of Infectious Diseases and Clinical Microbiology, Sivas Cumhuriyet University, Sivas, Türkiye; Faculty of Medicine, Department of Infectious Diseases and Microbiology, Istanbul Medipol University, Istanbul, Türkiye; Faculty of Medicine, Department of Infectious Diseases and Microbiology, Istanbul Medipol University, Istanbul, Türkiye; School of Medicine, Department of Infectious Diseases and Clinical Microbiology, University of Health Sciences, Kartal Dr. Lutfi Kirdar City Hospital, Istanbul, Türkiye; Department of Infectious Diseases and Clinical Microbiology, Erol Olçok Education and Research Hospital, Hitit University, Çorum, Türkiye; Department of Infectious Diseases and Clinical Microbiology, Erol Olçok Education and Research Hospital, Hitit University, Çorum, Türkiye; Faculty of Medicine, Department of Infectious Diseases and Microbiology, Başkent University, Ankara, Türkiye; Faculty of Medicine, Department of Infectious Diseases and Microbiology, Başkent University, Ankara, Türkiye; Faculty of Medicine, Department of Infectious Diseases and Microbiology, Dokuz Eylül University, İzmir, Türkiye; Faculty of Medicine, Department of Infectious Diseases and Clinical Microbiology, Erciyes University, Kayseri, Türkiye; Faculty of Medicine, Department of Infectious Diseases and Clinical Microbiology, Erciyes University, Kayseri, Türkiye; Department of Internal Medicine, Division of Intensive Care, Kayseri City Hospital, Kayseri, Türkiye; Department of Internal Medicine, Division of Intensive Care Medicine, Adana Seyhan State Hospital, Adana, Türkiye; Faculty of Medicine, Department of Infectious Diseases and Clinical Microbiology, Inönü University, Malatya, Türkiye; Faculty of Medicine, Department of Infectious Diseases and Clinical Microbiology, Amasya University Sabuncuoglu Serefeddin Training and Research Hospital, Amasya, Türkiye; Faculty of Medicine, Department of Infectious Diseases and Clinical Microbiology, Marmara University, Istanbul, Türkiye; Faculty of Medicine, Department of Infectious Diseases and Clinical Microbiology, Istanbul University, Çapa, Istanbul, Türkiye; Istanbul Faculty of Medicine, Department of Medical Microbiology, Istanbul University, Çapa, Istanbul, Türkiye; Faculty of Medicine, Department of Infectious Diseases and Clinical Microbiology, Istanbul University, Çapa, Istanbul, Türkiye; Faculty of Medicine, Department of Infectious Diseases and Clinical Microbiology, Mersin University, Mersin, Türkiye; Faculty of Medicine, Department of Infectious Diseases and Clinical Microbiology, Acıbadem Mehmet Ali Aydınlar University, Istanbul, Türkiye; Faculty of Medicine, Department of Medical Microbiology, Hacettepe University, Ankara, Türkiye; Faculty of Medicine, Department of Medical Microbiology, Amasya University Sabuncuoglu Serefeddin Training and Research Hospital, Amasya, Türkiye; Department of Medical Microbiology, Ankara City Hospital, Ankara, Türkiye; Faculty of Medicine, Department of Medical Microbiology, Uludağ University, Bursa, Türkiye; Faculty of Medicine, Department of Medical Microbiology, Çanakkale Onsekiz Mart University, Çanakkale, Türkiye; Faculty of Medicine, Department of Medical Microbiology, Selcuk University, Konya, Türkiye; School of Medicine, Department of Medical Microbiology, Pamukkale University, Denizli, Türkiye; Faculty of Medicine, Department of Medical Microbiology, Sivas Cumhuriyet University, Sivas, Türkiye; Faculty of Medicine, Department of Medical Microbiology, Dokuz Eylül University, İzmir, Türkiye; Department of Medical Microbiology, Kayseri City Hospital, Kayseri, Türkiye; Department of Medical Microbiology, Adana Seyhan State Hospital, Adana, Türkiye; Faculty of Medicine, Department of Medical Microbiology, Inönü University, Malatya, Türkiye; Faculty of Health, Medicine and Behavioural Sciences, UQ Centre for Clinical Research, University of Queensland, Brisbane, QLD, Australia; Faculty of Health, Medicine and Behavioural Sciences, UQ Centre for Clinical Research, University of Queensland, Brisbane, QLD, Australia; School of Medicine, Department of Infectious Diseases and Microbiology, Hacettepe University, Ankara, Türkiye; ADVANCE-ID, Saw Swee Hock School of Public Health, National University of Singapore, Singapore

## Abstract

**Background:**

Delays in appropriate antimicrobial therapy can increase the risk of all-cause mortality (ACM) in hospital-acquired bloodstream infections (HA-BSIs) caused by Gram-negative bacteria (GNB). We aimed to evaluate the effectiveness of early appropriate antimicrobial therapy (EAAT) compared with late appropriate antimicrobial therapy (LAAT) in this population.

**Methods:**

This study used data from a multi-centre, prospective cohort including adult patients with HA-BSI caused by GNB across 22 Turkish hospitals. A hypothetical target trial allocating participants with HA-BSI caused by monobacterial GNB to either EAAT or LAAT was emulated using the weighting approach. The primary outcome was 14 day ACM; 28 day ACM was a secondary outcome. Cox proportional hazards models with inverse probability weighting were applied to account for confounding, with antibiotic treatment incorporated as a time-dependent variable.

**Results:**

Among 680 patients, 14 day ACM occurred in 14.7% (40/272) of the EAAT group and 42.9% (175/408) of the LAAT group. EAAT was associated with a lower risk of 14 day ACM [adjusted hazard ratio (aHR) = 0.41; 95% CI: 0.28–0.61). Similarly, 26.1% (71/272) of the patients treated with EAAT and 49.5% (202/408) of those receiving LAAT died during 28 day follow-up (aHR = 0.66; 95% CI: 0.50–0.86). In the carbapenem-resistant GNB subset, EAAT reduced the hazard of 14 day ACM (aHR = 0.36; 95% CI: 0.21–0.60) and 28 day ACM (aHR = 0.60; 95% CI: 0.44–0.84). All prespecified and post hoc analyses consistently supported these findings.

**Conclusions:**

EAAT was associated with a survival benefit in individuals with HA-BSI due to GNB. Although these findings support early initiation of appropriate therapy, residual confounding cannot be excluded.

## Introduction

Hospital-acquired bloodstream infections (HA-BSIs) with Gram-negative bacteria (GNB) cause enormous burdens in disability-adjusted life years and are associated with high risk of morbidity and mortality.^1–3^ There are multiple patient and hospital-based determinants of adverse clinical outcomes in HA-BSI.^4,5^ Although these determinants have been the focus of numerous reports worldwide,^4,6^ the real impact of early appropriate antimicrobial therapy (EAAT; initiated within 24 h of blood culture sampling) on all-cause mortality remains controversial due to heterogeneous results in studies published so far.^3,7,8^

A target trial emulation framework as a method for causal inference from observational data can provide an alternative where pragmatic randomized controlled trials (RCTs) are not ethical to conduct.^9^ For example, pragmatic RCTs may not be suitable for appreciation of the benefit of EAAT compared with late appropriate antimicrobial therapy (LAAT) for the treatment of Gram-negative bacteraemia. In such a hypothetical pragmatic trial, it would not be ethical to assign a group of patients to the LAAT arm.

This study aimed to explore the causal effect of EAAT (≤24 h) versus LAAT (>24 h) on mortality in adult patients with hospital-acquired monobacterial BSI with GNB.

## Material and methods

### Data source and collection

We used data from a multicentre, prospective cohort study at 22 hospitals in Türkiye. Consecutive hospitalized adult patients (≥18 years of age) with an initial episode of documented bacterial HA-BSI (BSI occurring ≥48 h following hospital admission) were included from 1 September 2021 to 30 September 2022. In case of recurrent HA-BSI, only the first episode was included for the analysis. The case report form, including a complete list of variables and an overview of data collection and query process, is presented in the Supplementary material (available as Supplementary data at *JAC* Online).

### Study design

We emulated a hypothetical target trial in which adults with HA-BSI caused by monobacterial GNB received or did not receive at least one appropriate antimicrobial therapy (i.e. administration of at least one type of antibiotic to which the organism was not resistant *in vitro*) within the first 24 h of the onset of HA-BSI. Antibiotics were administered as per local and/or national guidelines of each centre (with the dose and frequency determined by renal function). The total duration of treatment was determined by the treating clinicians. Enrolled patients were followed up for 28 days.

This study followed the Strengthening the Reporting of Observational Studies in Epidemiology (STROBE) reporting guideline.^10^ An overview of the design decisions made in the observational analyses to emulate the target trial is shown in Table 1. The details of definitions used in this study are provided in the Supplementary material.

**Table 1.** The target trial and its emulation comparing early appropriate antibiotic treatment (≤24 h) with late initiation (>24 h) of appropriate antibiotic therapy in individuals with hospital-acquired bloodstream infection with Gram-negative bacteria

Protocol element	Target trial	Target trial emulation	Data source
Eligibility criteria	All adult (≥18 years) patients who have monobacterial BSI with a GNB occurring ≥48 h following hospital admission	Same as target trial	Electronic health record and patient charts
Exclusion criteria	Patients who have infection occurring <48 h after hospital admission	Same as target trial	Electronic health record and patient charts
	Polymicrobial infection	Same as target trial	Electronic health record and patient charts
	Pregnant patients	Same as target trial	Electronic health record and patient charts
	Patients on end-of-life care	Same as target trial	Electronic health record and patient charts
Treatment strategies	(1) Initiation of appropriate antibiotic therapy within 24 h of the onset of HA-BSI	Same as target trial	—
	(2) Initiation of appropriate antibiotic therapy >24 h after the onset of HA-BSI	Same as target trial	—
Treatment assignment	Randomization, no blinding	Eligible individuals were classified according to the treatment strategy consistent with their actual antibiotic administration on the index date (defined as Day 1 after the initial blood culture). Assignment was treated as if randomized within strata of baseline covariates, including age, gender, SOFA score, pre-admission health status, source of BSI, onset of BSI within 7 days of hospital admission, pathogen type, carbapenem resistance status, and Charlson comorbidity index	Electronic health record and patient charts
Follow-up	Starts at treatment assignment (Day 1) and ends at mortality or 28 days after baseline	Same as target trial	Electronic health record and patient charts
Outcomes	Primary: 14 day all-cause mortality	Same as target trial	Electronic health record and patient charts
	Secondary: 28 day all-cause mortality	Same as target trial	Electronic health record and patient charts
Causal estimand	Intention-to-treat effect	Observational analogue of modified intention-to-treat effect	—
Statistical analysis	Intention-to-treat analysis: Kaplan–Meier survival curves and Cox regression to estimate HRs	Inverse probability-weighted Kaplan–Meier survival curves and weighted Cox proportional hazards models, with antibiotic treatment incorporated as a time-dependent variable^a^	—

BSI, bloodstream infection; GNB, Gram-negative bacteria; HA-BSI, hospital-acquired bloodstream infection.

^a^Adjusted for age, gender, SOFA score, pre-admission health status, source of BSI, onset of BSI within 7 days of hospital admission, pathogen type, carbapenem resistance status and Charlson comorbidity index.

### Eligibility criteria

To meet eligibility criteria, patients had to have a monobacterial HA-BSI caused by GNB. Patients on end-of-life care, pregnant patients and those having community-acquired BSI or polymicrobial BSI (i.e. growth of multiple different microorganism species in the same blood culture) were excluded.

### Treatment strategies

The index day was defined as Day 1 after the initial blood culture yielding monobacterial GNB. Patients were categorized according to whether they received or did not receive appropriate antimicrobial therapy during the first 24 h of the onset of HA-BSI. Twenty-four hours was chosen as the period for treatment exposure since it is an achievable target with the use of MALDI-TOF for bacterial identification and detection of antimicrobial resistance determinants with molecular rapid diagnostic methods.^11,12^

### Follow-up and outcomes

Follow-up was initiated at index day (i.e. Day 1 after the first positive blood culture) and ended on Day 28, or the time of death, whichever occurred first. The primary outcome was 14 day all-cause mortality, while 28 day all-cause mortality was assessed as a secondary outcome.

### Microbiological studies

All causative bacteria identified in blood cultures of index HA-BSI episodes were collected in a central Turkish laboratory in Malatya, Türkiye (Inonu University Faculty of Medicine, molecular microbiology laboratory). Broth microdilution for colistin was performed on carbapenem-resistant GNB (CR-GNB) in a Turkish coordinating laboratory.^13^ GNB isolated from blood cultures in enrolled patients were submitted for further analysis at the coordinating laboratory in Brisbane, Australia (The University of Queensland Centre for Clinical Research). GN7F plates were used to perform broth microdilution (BMD) to determine MICs of a range of antibiotics according to manufacturers’ recommendations (Sensititre, ThermoFisher). Plates were read using the Sensititre manual viewer. Bacterial identification was confirmed by MALDI-TOF MS in both coordinating laboratories. Antibiotic susceptibility results were interpreted according to EUCAST breakpoints.^14^

If an organism was non-viable on subculture or a pure subculture could not be obtained, the site laboratory testing results were used to define antibiotic susceptibilities. Carbapenem resistance was defined as any isolate testing resistant to meropenem or imipenem (or ertapenem for *Enterobacterales*). For intrinsically imipenem-resistant species (e.g. *Proteus*, *Providencia* and *Morganella* spp.), ertapenem or meropenem testing results were used to define carbapenem resistance.

### Statistical analysis

A minimum of 362 patients were needed to have an 80% power with a two-sided 95% CI to detect a 12.5% difference in the primary outcome of 14 day all-cause mortality between treatment groups, assuming a 30% mortality rate in the LAAT group.^15^ The expected 14 day all-cause mortality was estimated from the results of the Eurobact II studies.^3,15^

**Figure 1.**
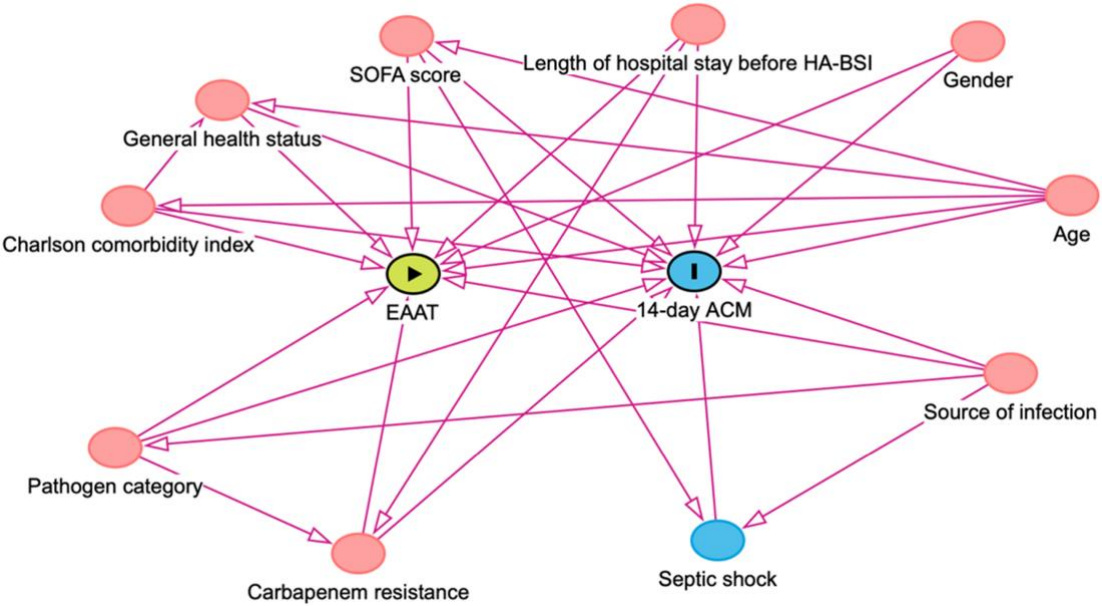
Identification of confounding variables using a directed acyclic graph (DAG). DAG was performed using the DAGitty v3.1 software (https://dagitty.net/dags.html). ACM, all-cause mortality; EAAT, early appropriate antimicrobial therapy; HA-BSI, hospital-acquired bloodstream infection.

Continuous data were presented as means with SDs. Categorical data were presented as frequencies and percentages. To emulate random assignment and achieve comparable results between treatment groups, we identified factors that independently associate with both treatment assignment and outcomes. We used a directed acyclic graph (DAG) to illustrate the causal system of interest in order to establish the model (Figure 1). Baseline covariates were chosen a priori, informed by clinical knowledge and assessed in the DAG. We adjusted for baseline confounders using inverse probability treatment weighting (IPTW) with pre-specified covariates: age, gender, general health status before admission, early-onset (within 7 days of hospital admission) versus late-onset BSI (beyond 7 days of hospital admission), source of BSI, type of pathogen, Charlson comorbidity index, carbapenem resistance status and SOFA score at the time of blood culture collection (Model A). To validate the balance of covariates after IPTW, standardized mean differences (SMDs) in confounder variables between treatment groups were calculated before and after weighting, with values <0.1 considered indicative of adequate balance.

Weighted Cox proportional hazards models were constructed to estimate HRs for the study outcomes. In these models, antibiotic treatment was specified as a time-dependent variable, allowing each patient's exposure status to vary over the follow-up period according to the timing of treatment initiation. This approach accounts for changes in treatment status over time and reduces the risk of bias associated with misclassification of exposure or immortal time bias. The validity of the analysis relies on the assumptions of no unmeasured confounding, positivity and correct specification of the IPTW model. Weights were truncated at the 99th percentile to limit the influence of extreme values and reduce the risk of model misspecification. Robust variance estimation with clustering on individual identifiers was used to account for within-subject correlation. The proportional hazards assumption was assessed using Schoenfeld residuals, and no substantial violations were detected. The E-value was also calculated to evaluate the robustness of association with potential unmeasured confounding factors.^16^

Weighted Kaplan–Meier survival curves were estimated to evaluate survival probabilities over time. Differences in survival between groups were assessed using a weighted log-rank test.

The effect size was estimated using the Stata command ‘teffects’, and ORs with 95% CIs were also calculated to compare 14 day and 28 day all-cause mortality between EAAT and LAAT in the main analysis population. Non-parametric bootstrapping with 200 samples was used to calculate 95% CI.

As there were no participants who deviated from their assigned treatment strategy, intention-to-treat (ITT) and per-protocol (PP) analyses would yield identical results. Therefore, we report a single analysis that is equivalent to both approaches.

All statistical analyses were conducted using STATA software, version 17 (StataCorp, TX, USA) and R v.4.0.2.

### Prespecified and post hoc analyses

We performed four prespecified and two post hoc analyses. First, the primary analysis examined EAAT defined as treatment within 24 h of the onset of HA-BSI. The second analysis adjusted the cut-off for defining EAAT to within 6 h of the onset of HA-BSI. For sensitivity analysis, we replaced the Charlson comorbidity index and SOFA score with immunosuppression status and the Pitt bacteraemia score (Model B). Given the difficulties in treating CR-GNB infections, a prespecified sensitivity analysis was conducted for patients with HA-BSI caused by CR-GNB. Lastly, as post hoc analyses, the causal effects of early appropriate therapy with type 2 carbapenems (i.e. monotherapy with meropenem or imipenem) versus late appropriate therapy with type 2 carbapenems on 14 day and 28 day all-cause mortality were examined. The same analyses were performed to estimate the causal effects of early polymyxin-based regimens compared with late polymyxin-based therapies on 14 day and 28 day all-cause mortality. Because of multiple comparisons, findings for sensitivity and post hoc analyses should be interpreted as exploratory.

### Missing data

We had complete data for all variables included in the main analysis.

### Ethics

This study was approved by the Hacettepe University Faculty of Medicine Clinical Research Ethics Committee (ref no. KA-21060), and informed consent was obtained from all patients and/or their legally authorized representatives.

## Results

Of 680 patients meeting the eligibility criteria, 272 (40%) were treated with EAAT, while 408 (60%) received LAAT. A flowchart of the patient inclusion process is shown in Figure S1.

The baseline characteristics of the patients are given in Table 2. The mean SOFA score was 7.0 (SD 4.55), and the great majority of the participants had a Charlson comorbidity score >2 (77%). Primary HA-BSI (i.e. presence of no clear source of infection or portal of entry) was the most common type of HA-BSI (40%), followed by those secondary to lower respiratory tract infections (22%) and central venous catheter–associated infections (14%). Source control was required for 29.3% of the patients, and was adequately achieved in 53.3% of these patients within 3 days of the onset of HA-BSI (Table 2). *Klebsiella* spp. was the most frequent causative pathogen (*n* = 229, 33.7%), followed by *Escherichia coli* (*n* = 149, 21.9%), and *Acinetobacter* spp. (*n* = 143, 21.0%). Three hundred and forty-one (50.1%) patients were infected by phenotypically carbapenem-resistant GNB, with a higher frequency in the LAAT group (67.6% versus 23.9%, *P* < 0.001). The characteristics of patients included in the CR-GNB subset are depicted in Table S1. Empirical and definitive antibiotic treatments prescribed for patients included in the main analysis population and the CR-GNB subset are shown in Tables S2 and S3, respectively.

**Table 2.** Baseline characteristics of the study population according to receipt of early appropriate antimicrobial therapy and late appropriate antimicrobial therapy

Characteristics	LAAT (>24 h) (*n* = 408)	EAAT (within 24 h) (*n* = 272)	*P* value	SMD (before weighted)	SMD^a^ (after weighted)	LAAT (>6 h) (*n* = 477)	EAAT (within 6 h) (*n* = 203)	*P* value	SMD (before weighted)	SMD (after weighted)
Admission source, *n* (%**)**			0.44					0.41		
Other hospital	39 (9.6)	19 (7.0)				43 (9.0)	15 (7.4)			
Home	353 (86.5)	244 (89.7)				414 (86.8)	183 (90.1)			
LTCF	16 (3.9)	9 (3.3)				20 (4.2)	5 (2.5)			
Age, mean (SD), y	64.87 ± 16.93	62.81 ± 17.79	0.13	0.118	0.004	64.66 ± 16.96	62.60 ± 18.02	0.15	0.118	0.011
Gender, male, *n* (%)	226 (55.4)	160 (58.8)	0.37	0.034	0.018	261 (54.7)	125 (61.6)	0.09	0.168	0.014
BMI, kg/m^2^, *n* (%)			0.76					0.76		
<18.5	10 (2.5)	9 (3.3)				12 (2.5)	7 (3.4)			
18.5–30	332 (81.4)	217 (79.8)				385 (80.7)	164 (80.8)			
>30	66 (16.2)	46 (16.9)				80 (16.8)	32 (15.8)			
CCI, *n* (%)			0.76	0.002	0.008			0.57	0.180	0.006
0	29 (7.1)	17 (6.3)				34 (7.1)	12 (5.9)			
1–2	63 (15.4)	47 (17.3)				73 (15.3)	37 (18.2)			
>2	316 (77.5)	208 (76.5)				370 (77.6)	154 (75.9)			
Severely restricted health status before hospital admission, *n* (%)	111 (27.2)	54 (19.9)	0.03	0.173	0.074	128 (26.8)	37 (18.2)	0.02	0.186	0.029
PBS, mean (SD)	4.6 ± 3.6	3.2 ± 3.4	<0.001	0.413	0.021	4.5 ± 3.6	3.0 ± 3.3	<0.001	0.426	0.062
SOFA score, mean (SD)	7.5 ± 4.6	6.2 ± 4.3	<0.001	0.293	0.036	7.5 ± 4.6	5.9 ± 4.1	<0.001	0.357	0.068
Septic shock, *n* (%)	154 (37.7)	70 (25.7)	0.001			175 (36.7)	49 (24.1)	0.001		
Residence in ICU, *n* (%)	275 (67.4)	146 (53.7)	<0.001			318 (66.7)	103 (50.7)	<0.001		
Receipt of vasopressors, *n* (%)	164 (40.2)	74 (27.2)	0.001			185 (38.8)	53 (26.1)	0.002		
Mechanical ventilation, *n* (%)	239 (58.6)	107 (39.3)	<0.001			273 (57.2)	73 (36.0)	<0.001		
Central venous catheters, *n* (%)	292 (71.6)	177 (65.1)	0.07			339 (71.1)	130 (64.0)	0.07		
Late-onset HA-BSI, *n* (%)	246 (60.3)	158 (58.1)	0.56	0.022	0.022	286 (60.0)	118 (58.1)	0.65	0.018	0.027
Source of BSI, *n* (%)			0.36	0.215	0.007			0.16	0.279	0.013
Primary	162 (39.7)	114 (41.9)	0.87			191 (40.0)	85 (41.9)	0.94		
Respiratory	101 (24.8)	51 (18.8)	0.06			118 (24.7)	34 (16.7)	0.02		
CVC	53 (13.0)	42 (15.4)	0.36			61 (12.8)	34 (16.7)	0.17		
Urinary	46 (11.3)	30 (11.0)	0.92			53 (11.1)	23 (11.3)	1.00		
Intra-abdominal	37 (9.1)	24 (8.8)	0.91			43 (9.0)	18 (8.9)	1.00		
Others	9 (2.2)	11 (4.0)	0.25			11 (2.3)	9 (4.4)	0.21		
Source control, *n* (%)			0.27					0.01		
Not required	293 (71.8)	188 (69.2)				345 (72.3)	136 (66.9)			
Required, achieved	55 (13.5)	51 (18.7)				63 (22.0)	43 (36.4)			
Required, but not achieved	60 (14.7)	33 (12.1)				69 (24.1)	24 (20.3)			
Type of pathogen, *n* (%)			**<**0.001	0.203	0.008			**<**0.001	0.273	0.013
*Escherichia coli*	49 (12.0)	100 (36.8)				70 (14.7)	79 (38.9)			
*Klebsiella* spp.	150 (36.8)	79 (29.0)				174 (36.5)	55 (27.1)			
*Acinetobacter* spp.	119 (29.2)	24 (8.8)				125 (26.2)	18 (8.9)			
*Pseudomonas* spp.	40 (9.8)	30 (11.0)				49 (10.3)	21 (10.3)			
Others	50 (12.2)	39 (14.3)				59 (12.3)	30 (14.7)			
CR-GNB, *n* (%)	276 (67.6)	65 (23.9)	**<**0.001	0.437	0.004	294 (61.6)	47 (23.2)	**<**0.001	0.384	0.003
Immunosuppression, *n* (%)	115 (28.2)	92 (33.8)	0.12	0.156	0.003	140 (29.4)	67 (33.0)	0.34	0.108	0.015
Chemotherapy	57 (14.0)	50 (18.4)	0.12			73 (15.3)	34 (16.7)	0.63		
Steroid therapy	38 (9.3)	14 (5.1)	0.06			43 (9.0)	9 (4.4)	0.06		
Absolute neutropenia	14 (3.4)	16 (5.9)	0.18			20 (4.2)	10 (4.9)	0.82		
Solid organ transplant	11 (2.7)	8 (2.9)	1.00			12 (2.5)	7 (3.4)	0.67		
AIDS	1 (0.2)	3 (1.1)	0.31			1 (0.2)	3 (1.5)	0.08		
Others	17 (4.2)	17 (6.2)	0.30			23 (4.8)	11 (5.4)	0.89		
Comorbidities, *n* (%)										
Heart failure (NYHA 3)	53 (13.0)	25 (9.2)	0.16			59 (12.4)	19 (9.4)	0.32		
Myocardial infarction	30 (7.4)	18 (6.6)	0.71			34 (7.1)	14 (6.9)	0.91		
Peripheral vascular disorder	23 (5.6)	17 (6.3)	0.87			27 (5.7)	13 (6.4)	0.84		
Cerebrovascular disorder	71 (17.4)	44 (16.2)	0.67			82 (17.2)	33 (16.3)	0.76		
Dementia	32 (7.8)	24 (8.8)	0.65			40 (8.4)	16 (7.9)	0.83		
Hemiplegia	16 (3.9)	9 (3.3)	0.83			18 (3.8)	7 (3.4)	1.00		
Uncomplicated DM	80 (19.6)	33 (12.1)	0.01			86 (18.0)	27 (13.3)	0.13		
Complicated DM	58 (14.2)	36 (13.2)	0.71			67 (14.0)	27 (13.3)	0.89		
Severe COPD	14 (3.4)	8 (2.9)	0.89			17 (3.6)	5 (2.5)	0.63		
Moderate COPD	43 (10.5)	23 (8.5)	0.37			49 (10.3)	17 (8.4)	0.53		
Connective tissue disorder	9 (2.2)	4 (1.5)	0.58			11 (2.3)	2 (1.0)	0.36		
Peptic ulcer	7 (1.7)	3 (1.1)	0.75			8 (1.7)	2 (1.0)	0.73		
Moderate or severe liver disease	5 (1.2)	5 (1.8)	0.53			6 (1.3)	4 (2.0)	0.49		
Chronic kidney disease	50 (12.3)	27 (9.9)	0.35			56 (11.7)	21 (10.3)	0.69		
Non-metastatic solid tumour	55 (13.5)	34 (12.5)	0.71			67 (14.0)	22 (10.8)	0.31		
Metastatic solid tumour	27 (6.6)	25 (9.2)	0.22			32 (6.7)	20 (9.9)	0.21		
Haematological malignancy	45 (11.0)	48 (17.6)	0.01			55 (11.5)	38 (18.7)	0.01		

CCI, Charlson comorbidity index; CR-GNB, carbapenem-resistant Gram-negative bacteria; CVC, central venous catheter; DM, diabetes mellitus; EAAT, early appropriate antimicrobial therapy; HA-BSI, hospital-acquired bloodstream infection; LAAT, late appropriate antimicrobial therapy; LTCF, long-term care facility; NYHA, New York Heart Association; PBS, Pitt bacteraemia score; SMD, standardized mean difference.

^a^Inverse probability weights were estimated and truncated at the 99th percentile to reduce the influence of extreme weights. The stabilized weights had a mean of 1.3 and variance of 0.7, with no evidence of extreme values. Covariate balance improved substantially after weighting, with all standardized mean differences below 0.1.

The distribution of the applied weights was acceptable, with a mean of 1.3 and a maximum of 3.2. After weighting, confounders were well balanced between groups, with no SMDs exceeding 0.1 (Tables 2 and S4).

### Primary outcome

Fourteen-day all-cause mortality occurred in 14.7% (40/272) of patients in the EAAT group and 42.9% (175/408) in the LAAT group. After adjustment using IPTW, EAAT resulted in a substantially lower hazard of 14 day mortality compared with LAAT, with adjusted hazard ratios (aHRs) of 0.41 (95% CI: 0.28–0.61; Table 3) in Model A and 0.45 (95% CI: 0.31–0.67) in Model B. Similar effects were observed among patients with CR-GNB infections, where EAAT significantly reduced the hazard of 14 day mortality (aHR = 0.36; 95% CI: 0.21–0.60; Table 3). For the main analysis population, the E-value was 4.31 for the estimate and 2.66 for the lower limit of CI, indicating that relatively weak unmeasured confounding associations could explain away the observed association.

**Table 3.** Primary and secondary outcomes among patients treated with EAAT or LAAT for monobacterial HA-BSI caused by GNB

All patients	EAAT (within 24 h), *n* (%)	LAAT (>24 h), *n* (%)	Adjusted HR (95% CI)^a^
14 day ACM	40 (14.7)	175 (42.9)	0.41 (0.28–0.61)
28 day ACM	71 (26.1)	202 (49.5)	0.66 (0.50–0.86)

All patients	EAAT (within 6 h), *n* (%)	LAAT (>6 h), *n* (%)	Adjusted HR (95% CI)^a^
14 day ACM	22 (10.8)	193 (40.5)	0.29 (0.17–0.49)
28 day ACM	45 (22.2)	228 (47.8)	0.61 (0.46–0.81)

Patients with CR-GNB infections	EAAT (within 24 h), *n* (%)	LAAT (>24 h), *n* (%)	Adjusted HR (95% CI)^a^
14 day ACM	18 (27.7)	145 (52.5)	0.36 (0.21–0.60)
28 day ACM	33 (50.8)	160 (58.0)	0.60 (0.44–0.84)

Patients with CR-GNB infections	EAAT (within 6 h), *n* (%)	LAAT (>6 h), *n* (%)	Adjusted HR (95% CI)^a^
14 day ACM	7 (14.9)	156 (53.1)	0.21 (0.09–0.44)
28 day ACM	20 (42.6)	173 (58.8)	0.48 (0.34–0.68)

Patients treated with carbapenems^b^	EAAT (within 24 h), *n* (%)	LAAT (>24 h), *n* (%)	Adjusted HR (95% CI)^a^
14 day ACM	9 (8.3)	25 (30.5)	0.28 (0.13–0.63)
28 day ACM	24 (22.0)	31 (37.8)	0.40 (0.23–0.70)

Patients treated with polymyxins^c^	EAAT (within 24 h), *n* (%)	LAAT (>24 h), *n* (%)	Adjusted HR (95% CI)^a^
14 day ACM	22 (40.0)	90 (55.6)	0.74 (0.44–1.24)
28 day ACM	30 (54.5)	101 (62.3)	0.98 (0.66–1.46)

ACM, all-cause mortality; EAAT, early appropriate antimicrobial therapy; GNB, Gram-negative bacteria; HA-BSI, hospital-acquired bloodstream infections; LAAT, late appropriate antimicrobial therapy.

^a^Adjusted for baseline confounders using inverse probability treatment weighting (IPTW) with pre-specified covariates: age, gender, general health status before admission, early-onset (within 7 days of hospital admission) versus late-onset BSI (beyond 7 days of hospital admission), source of BSI, type of pathogen, Charlson comorbidity index, carbapenem resistance status and SOFA score. In weighted Cox regression models, antimicrobial treatment was defined as a time-varying covariate.

^b^This denotes antimicrobial therapies containing meropenem or imipenem monotherapy (*n* = 191).

^c^This denotes antimicrobial regimens containing polymyxin monotherapy or polymyxin-containing combination regimens (*n* = 217).

For 14 day all-cause mortality with the 6 h threshold, the benefit with EAAT was substantial (10.8% versus 40.5%; Model A: aHR = 0.29; 95% CI: 0.17–0.49; Model B: aHR = 0.32; 95% CI: 0.19–0.53). The benefit with EAAT was also notable in the CR-GNB subgroup (aHR = 0.21; 95% CI: 0.09–0.44; Table 3).

Consistent with the results of time-to-death analyses, EAAT was associated with lower odds of 14 day all-cause mortality compared with LAAT when the EAAT threshold was defined as 24 h (aOR = 0.37; 95% CI: 0.23–0.58; Table S5) and 6 h (aOR = 0.26; 95% CI: 0.15–0.46; Table S5).

In post hoc analyses, patients treated with early carbapenem monotherapy had a significantly lower adjusted risk of mortality compared with those receiving late carbapenem monotherapy (aHR = 0.28; 95% CI: 0.13–0.63; Table 3). However, early polymyxin-based regimens did not confer significant mortality benefit over late polymyxin-based regimens (aHR = 0.74; 95% CI: 0.44–1.24; Table 3).

### Secondary outcome

**Figure 2.**
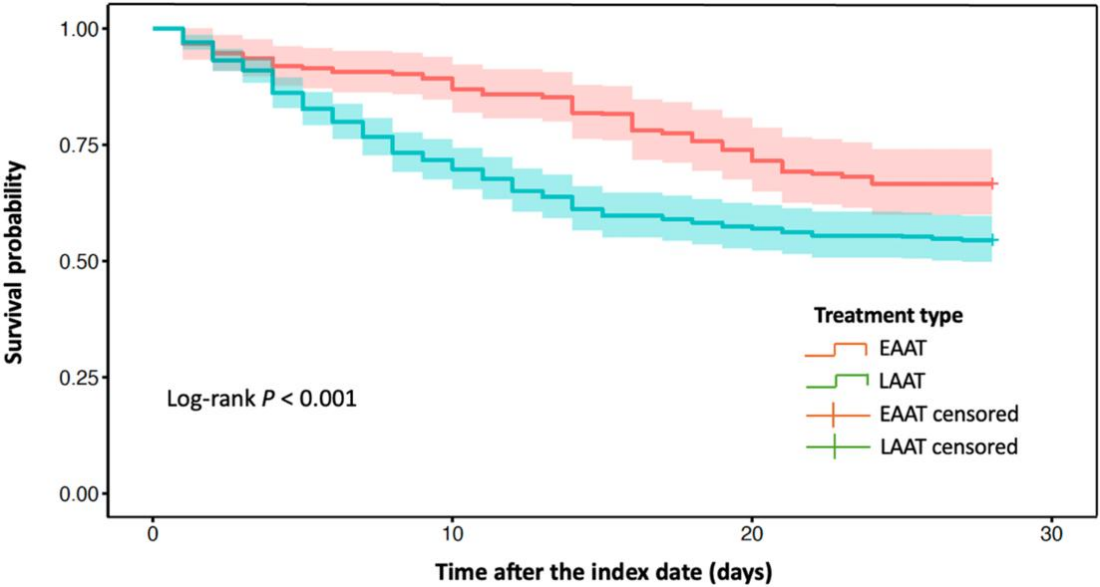
Weighted survival curves for patients receiving EAAT versus LAAT. EAAT, early appropriate antimicrobial therapy; LAAT, late appropriate antimicrobial therapy.

During the 28 day follow-up, 26.1% (71/272) of patients treated with EAAT and 49.5% (202/408) of those treated with LAAT died. After adjustment using IPTW, EAAT was associated with a lower hazard of 28 day all-cause mortality, with aHRs of 0.66 (95% CI: 0.50–0.86) in Model A and 0.71 (95% CI: 0.54–0.93) in Model B (Table 3). Weighted survival curves are shown in Figure 2. Among patients with CR-GNB infections, early therapy demonstrated a similar benefit to that observed in the main analysis population (aHR = 0.60; 95% CI: 0.44–0.84; Table 3).

When applying a 6 h threshold for early therapy, 28 day all-cause mortality showed a consistent pattern, remaining significantly lower in the EAAT group than in the LAAT group after IPTW adjustment using Model A (aHR = 0.61; 95% CI: 0.46–0.81; Table 3) and Model B (aHR = 0.63; 95% CI: 0.48–0.84). In the patients with CR-GNB infections, initiation of EAAT within 6 h of HA-BSI onset resulted in a significantly lower 28 day all-cause mortality compared with LAAT initiated beyond 6 h (aHR = 0.48; 95% CI: 0.34–0.68; Table 3).

In line with the time-to-death analyses, EAAT was associated with lower odds of 28 day all-cause mortality compared with LAAT when early therapy was defined using a 24 h threshold (aOR = 0.67; 95% CI: 0.45–0.98; Table S5) and a 6 h threshold (aOR = 0.62; 95% CI: 0.40–0.97; Table S5).

In post hoc analyses, patients who received carbapenem monotherapy within 24 h of HA-BSI onset had a significantly lower 28 day all-cause mortality compared with those who received LAAT (aHR = 0.40; 95% CI: 0.23–0.70; Table 3). In contrast, early initiation of polymyxin-based regimens did not provide a significant reduction in 28 day all-cause mortality compared with delayed initiation (aHR = 0.98; 95% CI: 0.66–1.46; Table 3).

## Discussion

In this multicentre, prospective cohort study, EAAT had a lower 14 day and 28 day mortality compared with those treated with LAAT among adult patients with monomicrobial HA-BSI caused by GNB. A similar pattern was observed in the patients with CR-GNB infections, where EAAT provided a clear survival advantage compared with LAAT. These findings were consistent across pre-specified sensitivity analyses, including those redefining EAAT as treatment initiated within 6 h of HA-BSI onset.

In post hoc analyses, early initiation of carbapenem therapy led to a substantial reduction in mortality, consistent with the findings from the overall cohort. In contrast, early use of polymyxin-based regimens did not lower 14 day or 28 day mortality compared with delayed initiation. This finding is consistent with prior evidence showing suboptimal clinical outcomes with polymyxin-based therapies despite *in vitro* susceptibility, likely related to unfavourable pharmacokinetic/pharmacodynamic (PK/PD) profiles and an increased risk of toxicity.^17^ Likewise, multiple observational studies have reported better outcomes with newer β-lactam/β-lactamase inhibitor combinations compared with conventional treatment approaches, which have predominantly relied on polymyxin-based regimens.^18,19^

Taken together, these findings highlight the clinical importance of prompt and effective antimicrobial therapy, particularly in settings with a high burden of CR-GNB infections. In Türkiye, where access to many novel agents approved by the FDA and/or the EMA remains limited (e.g. aztreonam/avibactam, cefiderocol, ceftolozane/tazobactam, and sulbactam/durlobactam), these results further emphasize the urgent need to improve availability of these newer antibiotics.^20,21^

A recent causal inference analysis from the EUROBACT II study showed a significant improvement in 28 day survival with initiation of appropriate antimicrobial therapy within 24 h of HA-BSI onset.^22^ However, in this study, patients with BSIs caused by Gram-positive bacteria and fungi were also included. Moreover, the results of the EUROBACT-II study cannot be extrapolated to non-ICU patients since only patients being treated in ICU were included in this study. Lastly, the EUROBACT II study included only patients who were still alive 1 day after blood culture sampling, which may introduce immortal time bias. In contrast, we excluded patients with BSIs caused by Gram-positive bacteria and fungi because they have biological properties different from infections caused by GNB and different treatment responses to currently available therapies. Furthermore, patients who were treated in ICUs or normal wards and received at least one dose of appropriate antibiotic therapy were included in our study. Finally, we regarded ‘antibiotic treatment’ as a time-dependent variable in the Cox proportional hazards models. This approach captures changes in treatment status over time and helps minimize bias arising from exposure misclassification and immortal time bias.

### Strengths and limitations

This study has several strengths. First, an RCT comparing EAAT versus LAAT in the setting of HA-BSI with GNB would be unethical. Therefore, we applied the principles of a pragmatic RCT to a prospective multicentre cohort study to minimize the risk of bias (e.g. indication and immortal time biases). Second, sensitivity analyses using different adjustment models (Model A using Charlson comorbidity index and SOFA score; Model B using immunosuppression status and Pitt bacteraemia score) and post hoc analyses yielded consistent results across all outcomes, supporting the robustness of our findings. Third, collection of highly detailed granular data and testing antimicrobial susceptibilities with gold standard methodology (i.e. BMD) allowed us to adjust for all potential confounding variables, leading to a more reliable estimation of the treatment effect. Fourth, efficient quality control checks and good communication between the coordinating centre and study sites ensured absence of missing data. Similarly, there was no loss to follow-up and no problem with adherence to the treatment assignment. Another strength of this study was using an objective primary outcome measure (i.e. 14 day mortality), offering advantages like minimizing bias, easy interpretation of treatment effect and prioritization of a patient-centred outcome.

Our study has certain limitations. First, since this was an observational study, some potential confounding factors may not have been adjusted for and/or measured, which may affect the validity of our estimates. In particular, potential unmeasured confounders may include centre-level factors (e.g. implementation of sepsis protocols, availability of local antimicrobial guidelines, and availability of infectious diseases specialists 24/7), clinician-level factors (e.g. early recognition of sepsis), the adequacy and timing of source control, pathogen-related characteristics (e.g. virulence), and aspects of antimicrobial therapy beyond ‘appropriateness’ (e.g. PK/PD target attainment and potential synergistic interactions between antibiotics used in combination regimens). Second, given the critical role of antimicrobial exposure in severe HA-BSI due to GNB, heterogeneity in treatment regimens represents an additional limitation of this study. For instance, differences between groups in the use of monotherapy versus combination therapy, as well as variations in dose optimization strategies—such as extended infusion protocols and PK/PD target attainment—may have contributed as unmeasured confounders. Third, our study enrolled patients in Türkiye. While the burden of CR-GNB is very high in Türkiye, it can be presumed that the spectrum of resistance mechanisms and/or patient characteristics could be different in other countries, thereby diminishing the generalizability of our results. Moreover, the long-term effect of EAAT could not be analysed and may have an impact on our results,^23^ given that 32% of patients were still resident in hospital at Day 28. Fifth, we did not collect data regarding therapeutic drug monitoring to check whether the adequate PK/PD targets of given antimicrobials were attained. However, the adequacy of the dose administered was assured during the quality control checking. Lastly, as in our study, when multiple comparisons are made without considering the interaction between them, the probability of making a type 1 error increases significantly.

### Conclusions

Our findings indicate that EAAT provides a survival advantage in patients with HA-BSI caused by GNB. These results support the broader implementation of rapid diagnostic testing in Turkish hospitals to facilitate earlier initiation of appropriate therapy. However, given the observational design and lack of randomization, the results remain susceptible to residual confounding and should be interpreted with appropriate caution.

## Supplementary Material

dkag161_Supplementary_Data

## Data Availability

Data will be made available upon request to the corresponding author.

## Transparency declarations

D.L.P. reports research grants from Shionogi, Pfizer, bioMérieux, Merck and Gilead. He has served on advisory boards or as a consultant to the AMR Action Fund, CARB-X, GARDP, Menarini, Pfizer, BioVersys, Aurobac and ADVANZ, and has received honoraria from Shionogi, Entasis/Innoviva, Pfizer, bioMérieux, Cepheid and Zuellig. P.N.A.H. reports a research grant from Gilead, has served on advisory boards for OpGen, Merck and Sandoz, and has received honoraria for lectures from OpGen, Sandoz, Pfizer and bioMérieux, paid to The University of Queensland. M.A. reports research support from Pfizer and Gilead. He was also a member of the Data Review Committee of GSK. A.T.A. reports payment from Pfizer for manuscript preparation, unrelated to this work. All other authors: none to declare.

## Supplementary data

Figure S1 and Tables S1 to S5 are available as Supplementary data at *JAC* Online.
