# Supplementary material for: Emulating a target trial of early compared with late initiation of appropriate antibiotic therapy for hospital-acquired monobacterial Gram-negative bloodstream infections
Source: J Antimicrob Chemother. 2026 May 13;81(6):dkag161. doi: 10.1093/jac/dkag161 (PMC13171036; doi:10.1093/jac/dkag161)
Supplement: dkag161_Supplementary_Data [file dkag161_supplementary_data.docx]

**Online supplementary material**

**Emulating a Target Trial of Early Compared to Late Initiation of Appropriate Antibiotic Therapy for Hospital-acquired Monobacterial Gram-negative Bloodstream Infections**

Abdullah T. Aslan^1,2*^, Yukiko Ezure^1^, Elif Seren Tanrıverdi^3^, Osman Dağ^4^, Cansu Cimen^5^, Ayşe Kaya Kalem^6^, Bircan Kayaaslan^6^, Sevil Alkan^7^, Bahadır Köylü^8^, Emine Büşra Ata^8^, Bilge Çağlar^9^, Neşe Saltoğlu^9^, Uğur Önal^10^, Seçil Deniz^11^, Onur Ural^12^, Murtaza Öz^13^, Mehmet Bakır^13^, Mesut Yılmaz^14^, Rümeysa Çakmak^14^, Ayşe Batırel^15^, Özlem Akdoğan^16^, Nurcan Baykam^16^, Çiğdem Erol^17^, Tuğba Yanık Yalçın^17^, Oya Özlem Kutlusoy^18^, Zeynep Türe Yüce^19^, Gamze Kalın Ünüvar^19^, Zuhal Özer Şimşek^20^, Seda Güzeldağ^21^, Adem Köse^22^, Mustafa Cihangiroğlu^23^, Dilek Yağcı-Caglayik^24^, Muhammed Burak Sevinç^25^, Zerrin Aktaş^26^, Oral Öncül^25^, Gülden Ersöz^27^, Ayşe Sesin Kocagöz^28^, Gülşen Hazırolan^29^, Tuğçe Ünalan-Altıntop^30^, Bedia Dinç^31^, Nazmiye Ülkü Tüzemen^32^, Alper Akçalı^33^, Salih Maçin^34^, Ahmet Çalışkan^35^, Mürşit Hasbek^36^, Cem Ergon^37^, Yasemin Ay Altıntop^38^, Müge Şimşek^39^, Barış Otlu^3^, Kay A. Ramsay^1^, Patrick N.A. Harris^1^, Murat Akova^40^, David L. Paterson^41^, on behalf of the Study Group for Carbapenem Resistance (SCARE)

^1^Faculty of Health, Medicine and Behavioural Sciences, UQ Centre for Clinical Research, University of Queensland, Brisbane, QLD, Australia; ^2^Sunshine Coast University Hospital, Department of General Medicine, Sunshine Coast, QLD, Australia; ^3^Faculty of Medicine, Department of Medical Microbiology, Inönü University, Malatya, Türkiye; ^4^Faculty of Medicine, Department of Biostatistics, Hacettepe University, Ankara, Türkiye; ^5^Department of General Internal Medicine, Infectious Diseases and Tropical Medicine, Antwerp University Hospital, Antwerp, Belgium; ^6^Ankara Yildirim Beyazit University, Faculty of Medicine, Ankara Bilkent City Hospital, Department of Infectious Diseases and Clinical Microbiology, Ankara, Türkiye; ^7^Faculty of Medicine, Department of Infectious Diseases and Clinical Microbiology, Çanakkale Onsekiz Mart University, Çanakkale, Türkiye; ^8^School of Medicine, Department of Internal Medicine, Hacettepe University, Ankara, Türkiye; ^9^Cerrahpaşa Medical Faculty, Department of Infectious Diseases and Clinical Microbiology, Istanbul University, Istanbul, Türkiye; ^10^Faculty of Medicine, Department of Infectious Diseases and Clinical Microbiology, Uludağ University, Bursa, Türkiye; ^11^School of Medicine, Department of Infectious Diseases and Clinical Microbiology, Pamukkale University, Denizli, Türkiye; ^12^Faculty of Medicine, Department of Infectious Diseases and Clinical Microbiology, Selçuk University, Konya, Türkiye; ^13^Faculty of Medicine, Department of Infectious Diseases and Clinical Microbiology, Sivas Cumhuriyet University, Sivas, Türkiye; ^14^Faculty of Medicine, Department of Infectious Diseases and Microbiology, Istanbul Medipol University, Istanbul, Türkiye; ^15^School of Medicine, Department of Infectious Diseases and Clinical Microbiology, University of Health Sciences, Kartal Dr. Lutfi Kirdar City Hospital, Istanbul, Türkiye; ^16^Department of Infectious Diseases and Clinical Microbiology, Erol Olçok Education and Research Hospital, Hitit University, Çorum, Türkiye; ^17^Faculty of Medicine, Department of Infectious Diseases and Microbiology, Başkent University, Ankara, Türkiye; ^18^Faculty of Medicine, Department of Infectious Diseases and Microbiology, Dokuz Eylül University, İzmir, Türkiye; ^19^Faculty of Medicine, Department of Infectious Diseases and Clinical Microbiology, Erciyes University, Kayseri, Türkiye; ^20^Department of Internal Medicine, Division of Intensive Care, Kayseri City Hospital, Kayseri, Türkiye; ^21^Department of Internal Medicine, Division of Intensive Care Medicine, Adana Seyhan State Hospital, Adana, Türkiye; ^22^Faculty of Medicine, Department of Infectious Diseases and Clinical Microbiology, Inönü University, Malatya, Türkiye; ^23^Faculty of Medicine, Department of Infectious Diseases and Clinical Microbiology, Amasya University Sabuncuoglu Serefeddin Training and Research Hospital, Amasya, Türkiye; ^24^Faculty of Medicine, Department of Infectious Diseases and Clinical Microbiology, Marmara University, Istanbul, Türkiye; ^25^Faculty of Medicine, Department of Infectious Diseases and Clinical Microbiology, Istanbul University, Çapa, Istanbul, Türkiye; ^26^Istanbul Faculty of Medicine, Department of Medical Microbiology, Istanbul University, Çapa, Istanbul, Türkiye; ^27^Faculty of Medicine, Department of Infectious Diseases and Clinical Microbiology, Mersin University, Mersin, Türkiye; ^28^Faculty of Medicine, Department of Infectious Diseases and Clinical Microbiology, Acıbadem Mehmet Ali Aydınlar University, Istanbul, Türkiye; ^29^Faculty of Medicine, Department of Medical Microbiology, Hacettepe University, Ankara, Türkiye; ^30^Faculty of Medicine, Department of Medical Microbiology, Amasya University Sabuncuoglu Serefeddin Training and Research Hospital, Amasya, Türkiye; ^31^Department of Medical Microbiology, Ankara City Hospital, Ankara, Türkiye; ^32^Faculty of Medicine, Department of Medical Microbiology, Uludağ University, Bursa, Türkiye; ^33^Faculty of Medicine, Department of Medical Microbiology, Çanakkale Onsekiz Mart University, Çanakkale, Türkiye; ^34^Faculty of Medicine, Department of Medical Microbiology, Selcuk University, Konya, Türkiye; ^35^School of Medicine, Department of Medical Microbiology, Pamukkale University, Denizli, Türkiye; ^36^Faculty of Medicine, Department of Medical Microbiology, Sivas Cumhuriyet University, Sivas, Türkiye; ^37^Faculty of Medicine, Department of Medical Microbiology, Dokuz Eylül University, İzmir, Türkiye; ^38^Department of Medical Microbiology, Kayseri City Hospital, Kayseri, Türkiye; ^39^Department of Medical Microbiology, Adana Seyhan State Hospital, Adana, Türkiye; ^40^School of Medicine, Department of Infectious Diseases and Microbiology, Hacettepe University, Ankara, Türkiye; ^41^ADVANCE-ID, Saw Swee Hock School of Public Health, National University of Singapore, Singapore.

*Corresponding author: Abdullah T. Aslan

Address: The University of Queensland Centre for Clinical Research, Faculty of Medicine, Building 71/918 RBWH Herston, Brisbane City QLD 4006

Email: a.aslan@uq.edu.au

Phone: (07) 3346 5555

Fax: (07) 3346 5598

**Definitions**

**Admission source**: refers to where was the patient before hospital admission

**HA-BSI**: Presence of clinical signs and symptoms of BSI along with isolation of a GNB in blood culture sampled 48 h or more after hospital admission

**Appropriate antimicrobial therapy**: Prescription of at least one intravenous antibiotic in which the causative bacteria are not resistant *in vitro* according to EUCAST breakpoints.^1^ The adequacy of antimicrobial selection and dosing regimens were manually reviewed for all patients.

**Sources of HA-BSI**: They were defined by clinical likelihood assessment of the treating clinician each center. Primary HA-BSI denotes no clear source of infection or portal of entry.

**Source control**: It was reported according to the source and intervention, with adequacy assessed in collaboration with local investigators. The consistencies between the recorded infectious source, source control intervention and microbiology results were ensured via discussion with local investigators. Source control interventions were classified into three categories: 1) repair, diversion, or excision of an ongoing source of contamination, such as a perforated viscus; 2) drainage of fluid collections and abscesses; and 3) removal of foreign bodies colonized or infected by pathogens or the debridement of infected and devitalized tissues. Source control interventions undertaken beyond 72h of the onset of HA-BSI were considered inadequate.

**Glasgow coma scale (GCS)**: was entered as the lowest GCS over the last 24 hours of HA-BSI for non-sedated patients. For patients sedated, the GCS at the time of just prior to sedation was recorded.

**SOFA/modified SOFA**: The SOFA score was calculated at the onset of HA-BSI and at day 14.^2^ For patients who had been discharged at day 14, the SOFA score was presumed to have improved as per the secondary outcome measure. The worst parameter measured in the prior 24 hours was used while assessing the SOFA score. In non-ICU patients, modified SOFA score was calculated.^3^

**Earl-onset HA-BSI**: Less than 7 days between hospital admission and HA-BSI

**Late-onset HA-BSI**: ≥ 7 days between hospital admission and HA-BSI

**Data collection and quality processes**

Site investigators collected data by using hospital registries and patient charts and entered these data in a secure online database. Adherence checks of patients discharged from the hospital after switching to oral antimicrobial therapy were carried out by site investigators via telephone interviews. A training video providing detailed information about the study and standardised data entry procedures was shared with each investigator. A query process was used, which involved manual checking of each case report form (CRF) by a group of principal investigators (ATA, EST and CC) for data quality, logic and completeness. Any query was forwarded to the site investigator and rechecked until satisfactorily addressed. In the event of no response, a minimum of 3 attempts were made to contact the site investigator. Antibiotics administered were reviewed for appropriateness of dose, frequency and route of administration

**Table S1. Baseline characteristics and outcomes of patients included in CR-GNB subset**

| Characteristics | LAAT (>24h)  (n = 276) | EAAT (within 24h)  (n = 65) | *P*-value |
| --- | --- | --- | --- |
| Admission source, n (%) |  |  | 0.67 |
| Other hospital | 27 (9.8) | 7 (10.8) |  |
| Home | 235 (85.1) | 53 (81.5) |  |
| LTCF | 14 (5.1) | 5 (7.7) |  |
| Age, years, mean (SD) | 64.5±16.6 | 62.2±19.9 | 0.37 |
| Gender, male, n (%) | 153 (55.4) | 43 (66.2) | 0.15 |
| BMI, kg/m^2^, n (%) |  |  | 0.72 |
| <18.5 | 6 (2.2) | 1 (1.5) |  |
| 18.5-30 | 217 (78.6) | 54 (83.1) |  |
| >30 | 53 (19.2) | 10 (15.4) |  |
| CCI |  |  | 0.36 |
| 0 | 20 (7.2) | 7 (10.8) |  |
| 1-2 | 46 (16.7) | 7 (10.8) |  |
| >2 | 210 (76.1) | 51 (78.5) |  |
| Severely restricted health status before hospital admission, n (%) | 86 (31.2) | 19 (29.2) | 0.88 |
| PBS, mean (SD) | 5.3±3.5 | 5.2±3.6 | 0.87 |
| SOFA score, mean (SD) | 8.4±4.6 | 9.3±4.7 | 0.16 |
| Septic shock, n (%) | 125 (45.3) | 31 (47.7) | 0.73 |
| Residence in ICU, n (%) | 212 (76.8) | 49 (75.4) | 0.93 |
| Receipt of vasopressors, n (%) | 130 (47.1) | 32 (49.2) | 0.76 |
| Mechanical ventilation, n (%) | 189 (68.5) | 42 (64.6) | 0.55 |
| Central venous catheter, n (%) | 216 (78.3) | 53 (81.5) | 0.68 |
| Late onset HA-BSI, n (%) | 167 (60.5) | 44 (67.7) | 0.35 |
| Source of BSI, n (%) |  |  |  |
| Primary | 109 (39.5) | 21 (32.3) | 0.53 |
| Respiratory | 82 (29.7) | 23 (35.4) | 0.46 |
| CVC | 39 (14.1) | 8 (12.3) | 0.85 |
| Urinary | 21 (7.6) | 1 (1.5) | 0.09 |
| Intra-abdominal | 21 (7.6) | 8 (12.3) | 0.33 |
| Others | 4 (1.4) | 4 (6.2) | 0.05 |
| Source control, n (%) |  |  | 0.17 |
| Not required | 203 (73.5) | 43 (66.2) |  |
| Required, achieved | 35 (12.7) | 15 (23.1) |  |
| Required, but not achieved | 38 (13.8) | 7 (10.7) |  |
| Type of pathogen, n (%) |  |  | 0.01 |
| Escherichia coli | 6 (2.2) | 5 (7.7) |  |
| Klebsiella spp. | 107 (38.8) | 22 (33.8) |  |
| Acinetobacter spp. | 115 (41.7) | 20 (30.8) |  |
| Pseudomonas spp. | 18 (6.5) | 11 (16.9) |  |
| Others | 30 (10.8) | 7 (10.8) |  |
| Immunosuppression, n (%) | 77 (27.9) | 19 (29.2) | 0.95 |
| Chemotherapy | 37 (13.4) | 7 (10.8) | 0.71 |
| Steroid | 27 (9.8) | 5 (7.7) | 0.81 |
| Absolute neutropenia | 7 (2.5) | 3 (4.6) | 0.41 |
| Solid organ transplant | 8 (2.9) | 2 (3.1) | 1.00 |
| AIDS | 1 (0.4) | 2 (3.1) | 0.09 |
| Others | 10 (3.6) | 6 (9.2) | 0.11 |
| Comorbidities, n (%) |  |  |  |
| Heart failure | 37 (13.4) | 6 (9.2) | 0.48 |
| Myocardial infarction | 22 (8.0) | 3 (4.6) | 0.44 |
| Peripheral vascular disorder | 15 (5.4) | 2 (3.1) | 0.75 |
| Cerebrovascular disorder | 53 (19.2) | 12 (18.5) | 1.00 |
| Dementia | 19 (6.9) | 10 (15.4) | 0.05 |
| Hemiplegia | 12 (4.3) | 4 (6.2) | 0.52 |
| Uncomplicated DM | 58 (21.0) | 9 (13.8) | 0.26 |
| Complicated DM | 41 (14.9) | 7 (10.8) | 0.51 |
| Severe COPD | 8 (2.9) | 4 (6.2) | 0.25 |
| Moderate COPD | 33 (12.0) | 7 (10.8) | 0.96 |
| Connective tissue disorder | 8 (2.9) | 0 (0.0) | 0.36 |
| Peptic ulcer | 6 (2.2) | 1 (1.5) | 1.00 |
| Moderate or severe liver disease | 5 (1.8) | 2 (3.1) | 0.62 |
| Chronic kidney disease | 34 (12.3) | 10 (15.4) | 0.65 |
| Non-metastatic solid tumor | 36 (13.0) | 9 (13.8) | 1.00 |
| Metastatic solid tumor | 16 (5.8) | 3 (4.6) | 1.00 |
| Haematological malignancy | 28 (10.1) | 11 (16.9) | 0.18 |
| Outcomes |  |  |  |
| 14-day mortality | 145 (52.5) | 18 (27.7) | 0.001 |
| 28-day mortality | 160 (58.0) | 33 (50.8) | 0.29 |

Abbreviations: LAAT, late appropriate antimicrobial therapy; EAAT, early appropriate antimicrobial therapy; SMD, standardized mean differences; LTCF, long-term care facility; SD, standard deviation; BMI, body mass index; CCI, Charlson comorbidity index; PBS, Pitt Bacteremia Score, SOFA, Sequential Organ Failure Assessment; HA-BSI, hospital-acquired bloodstream infection; ICU, intensive care unit; CVC, central venous catheter; CR-GNB, carbapenem-resistant Gram-negative bacteria; NYHA, New York Hearth Association; DM, diabetes mellitus; COPD, chronic obstructive pulmonary disease.

**Table S2. Antimicrobial agents used in main analysis population**

| Main analysis population (N = 680) | |
| --- | --- |
| Empirical treatment |  |
| Carbapenem-based regimens | **315** |
| Meropenem monotherapy | 207 |
| Imipenem monotherapy | 24 |
| Ertapenem monotherapy | 10 |
| Carbapenem plus other antibiotics | 74 |
| Meropenem plus amikacin | 24 |
| Meropenem plus fosfomycin | 19 |
| Meropenem plus tigecycline | 16 |
| Meropenem plus ertapenem | 5 |
| Meropenem plus trimethoprim/sulfamethoxazole | 3 |
| Meropenem plus ampicillin-sulbactam | 2 |
| Meropenem plus ciprofloxacin | 1 |
| Meropenem plus fosfomycin plus amikacin | 1 |
| Meropenem plus cefoperazone-sulbactam | 1 |
| Imipenem plus levofloxacin | 1 |
| Ertapenem plus amikacin | 1 |
| Piperacillin-tazobactam-based regimens | **144** |
| Piperacillin-tazobactam monotherapy | 124 |
| Piperacillin-tazobactam plus other antibiotics | 20 |
| Piperacillin-tazobactam plus amikacin | 10 |
| Piperacillin-tazobactam plus tigecycline | 4 |
| Piperacillin-tazobactam plus ciprofloxacin | 2 |
| Piperacillin-tazobactam plus fosfomycin | 2 |
| Piperacillin-tazobactam plus gentamicin | 1 |
| Piperacillin-tazobactam plus levofloxacin | 1 |
| Polymyxins-based regimens | **111** |
| Polymyxin monotherapy | 12 |
| Polymyxin plus other antibiotics | 99 |
| Polymyxin plus meropenem | 58 |
| Polymyxin plus tigecycline | 13 |
| Polymyxin plus fosfomycin | 5 |
| Polymyxin plus cefoperazone-sulbactam | 5 |
| Polymyxin plus meropenem plus amikacin | 4 |
| Polymyxin plus tigecycline plus meropenem | 4 |
| Polymyxin plus fosfomycin plus meropenem | 3 |
| Polymyxin plus imipenem | 3 |
| Polymyxin plus trimethoprim/sulfamethoxazole | 1 |
| Polymyxin plus cefepime | 1 |
| Polymyxin plus cefepime plus tigecycline | 1 |
| Polymyxin plus levofloxacin | 1 |
| Ceftazidime-avibactam-based regimens | **9** |
| Ceftazidime-avibactam monotherapy | 3 |
| Ceftazidime-avibactam plus other antibiotics | 6 |
| Ceftazidime-avibactam plus fosfomycin | 2 |
| Ceftazidime-avibactam plus imipenem | 2 |
| Ceftazidime-avibactam plus polymyxin plus amikacin | 1 |
| Ceftazidime-avibactam plus tigecycline | 1 |
| Other antibiotic regimens | **101** |
| Ceftriaxone monotherapy | 31 |
| Ampicillin-sulbactam monotherapy | 12 |
| Ceftazidime monotherapy | 12 |
| Trimethoprim/sulfamethoxazole monotherapy | 7 |
| Levofloxacin monotherapy | 7 |
| Cefepime monotherapy | 7 |
| Cefepime plus amikacin | 5 |
| Tigecycline monotherapy | 4 |
| Ciprofloxacin monotherapy | 2 |
| Fosfomycin plus amikacin | 2 |
| Ceftazidime plus amikacin | 1 |
| Ceftriaxone plus levofloxacin | 1 |
| Ampicillin-sulbactam plus levofloxacin | 1 |
| Cefepime plus fosfomycin | 1 |
| Fosfomycin plus gentamicin | 1 |
| Cefepime plus trimethoprim/sulfamethoxazole | 1 |
| Fosfomycin monotherapy | 1 |
| Trimethoprim/sulfamethoxazole plus fosfomycin | 1 |
| Tigecycline plus cefoperazon-sulbactam | 1 |
| Tigecycline plus trimethoprim/sulfamethoxazole | 1 |
| Fosfomycin plus cefoperazone-sulbactam | 1 |
| Penicillin G | 1 |
| Definitive treatment (n = 638) | |
| Carbapenem-based regimens | **235** |
| Meropenem monotherapy | 140 |
| Imipenem monotherapy | 13 |
| Ertapenem monotherapy | 11 |
| Carbapenem plus other antibiotics | 71 |
| Meropenem plus amikacin | 26 |
| Meropenem plus fosfomycin | 18 |
| Meropenem plus tigecycline | 11 |
| Meropenem plus trimethoprim/sulfamethoxazole | 4 |
| Meropenem plus ertapenem | 2 |
| Meropenem plus tigecycline plus trimethoprim/sulfamethoxazole | 2 |
| Meropenem plus ampicillin-sulbactam | 1 |
| Meropenem plus gentamicin | 1 |
| Meropenem plus fosfomycin plus amikacin | 1 |
| Imipenem plus gentamicin | 1 |
| Meropenem plus ertapenem plus tigecycline | 1 |
| Imipenem plus amikacin | 1 |
| Imipenem plus amikacin plus trimethoprim/sulfamethoxazole | 1 |
| Meropenem plus tigecycline plus fosfomycin | 1 |
| Piperacillin-tazobactam-based regimens | **60** |
| Piperacillin-tazobactam monotherapy | 53 |
| Piperacillin-tazobactam plus other antibiotics | 7 |
| Piperacillin-tazobactam plus amikacin | 3 |
| Piperacillin-tazobactam plus tigecycline | 1 |
| Piperacillin-tazobactam plus ciprofloxacin | 1 |
| Piperacillin-tazobactam plus fosfomycin | 1 |
| Piperacillin-tazobactam plus trimethoprim/sulfamethoxazole | 1 |
| Polymyxins-based regimens | **187** |
| Polymyxin monotherapy | 45 |
| Polymyxin plus other antibiotics | 142 |
| Polymyxin plus meropenem | 79 |
| Polymyxin plus tigecycline | 17 |
| Polymyxin plus fosfomycin | 13 |
| Polymyxin plus tigecycline plus meropenem | 7 |
| Polymyxin plus ampicillin-sulbactam | 5 |
| Polymyxin plus imipenem | 5 |
| Polymyxin plus trimethoprim/sulfamethoxazole | 3 |
| Polymyxin plus fosfomycin plus meropenem | 3 |
| Polymyxin plus cefoperazone-sulbactam | 2 |
| Polymyxin plus trimethoprim/sulfamethoxazole plus fosfomycin | 2 |
| Polymyxin plus cefepime | 2 |
| Polymyxin plus imipenem | 1 |
| Polymyxin plus fosfomycin plus cefoperazone-sulbactam | 1 |
| Polymyxin plus ertapenem plus amikacin | 1 |
| Polymyxin plus ertapenem plus meropenem | 1 |
| Ceftazidime-avibactam-based regimens | **33** |
| Ceftazidime-avibactam monotherapy | 18 |
| Ceftazidime-avibactam plus other antibiotics | 15 |
| Ceftazidime-avibactam plus amikacin | 5 |
| Ceftazidime-avibactam plus fosfomycin | 2 |
| Ceftazidime-avibactam plus polymyxin plus fosfomycin | 2 |
| Ceftazidime-avibactam plus tigecycline | 2 |
| Ceftazidime-avibactam plus imipenem | 1 |
| Ceftazidime-avibactam plus amikacin plus fosfomycin | 1 |
| Ceftazidime-avibactam plus polymyxin | 1 |
| Ceftazidime-avibactam plus amikacin plus tigecycline | 1 |
| Other antibiotic regimens | **123** |
| Ceftriaxone monotherapy | 35 |
| Levofloxacin monotherapy | 16 |
| Trimethoprim/sulfamethoxazole monotherapy | 15 |
| Fosfomycin monotherapy | 15 |
| Ciprofloxacin monotherapy | 9 |
| Ceftazidime monotherapy | 6 |
| Cefepime monotherapy | 6 |
| Amikacin monotherapy | 4 |
| Amoxicillin-clavulanic acid monotherapy | 3 |
| Cefepime plus amikacin | 2 |
| Fosfomycin plus amikacin | 2 |
| Cefoperazon-sulbactam monotherapy | 2 |
| Trimethoprim/sulfamethoxazole plus fosfomycin | 2 |
| Tigecycline monotherapy | 1 |
| Ampicillin-sulbactam monotherapy | 1 |
| Ceftazidime plus amikacin | 1 |
| Levofloxacin plus trimethoprim/sulfamethoxazole | 1 |
| Tigecycline plus fosfomycin | 1 |
| Penicillin G | 1 |

42 patients did not receive any definitive antibiotic therapy

**Table S3. Antimicrobial agents used in carbapenem-resistant Gram-negative bacterial infections**

| Empirical treatment (n = 341) |  |
| --- | --- |
| Carbapenem-based regimens | **168** |
| Meropenem monotherapy | 94 |
| Imipenem monotherapy | 16 |
| Ertapenem monotherapy | 3 |
| Carbapenem plus other antibiotics | 55 |
| Meropenem plus amikacin | 18 |
| Meropenem plus fosfomycin | 14 |
| Meropenem plus tigecycline | 12 |
| Meropenem plus ertapenem | 3 |
| Meropenem plus ampicillin-sulbactam | 2 |
| Meropenem plus trimethoprim/sulfamethoxazole | 1 |
| Meropenem plus ciprofloxacin | 1 |
| Meropenem plus fosfomycin plus amikacin | 1 |
| Meropenem plus cefoperazone-sulbactam | 1 |
| Imipenem plus levofloxacin | 1 |
| Ertapenem plus amikacin | 1 |
| Piperacillin-tazobactam-based regimens | **49** |
| Piperacillin-tazobactam monotherapy | 39 |
| Piperacillin-tazobactam plus other antibiotics | 10 |
| Piperacillin-tazobactam plus amikacin | 4 |
| Piperacillin-tazobactam plus tigecycline | 3 |
| Piperacillin-tazobactam plus ciprofloxacin | 1 |
| Piperacillin-tazobactam plus fosfomycin | 1 |
| Piperacillin-tazobactam plus levofloxacin | 1 |
| Polymyxins-based regimens | **77** |
| Polymyxin monotherapy | 10 |
| Polymyxin plus other antibiotics | 67 |
| Polymyxin plus meropenem | 41 |
| Polymyxin plus tigecycline | 11 |
| Polymyxin plus meropenem plus amikacin | 3 |
| Polymyxin plus fosfomycin | 2 |
| Polymyxin plus tigecycline plus meropenem | 2 |
| Polymyxin plus fosfomycin plus meropenem | 2 |
| Polymyxin plus cefoperazone-sulbactam | 1 |
| Polymyxin plus imipenem | 1 |
| Polymyxin plus trimethoprim/sulfamethoxazole | 1 |
| Polymyxin plus cefepime | 1 |
| Polymyxin plus cefepime plus tigecycline | 1 |
| Polymyxin plus levofloxacin | 1 |
| Ceftazidime-avibactam-based regimens | **8** |
| Ceftazidime-avibactam monotherapy | 3 |
| Ceftazidime-avibactam plus other antibiotics | 5 |
| Ceftazidime-avibactam plus fosfomycin | 2 |
| Ceftazidime-avibactam plus imipenem | 2 |
| Ceftazidime-avibactam plus polymyxin plus amikacin | 1 |
| Other antibiotic regimens | **39** |
| Ampicillin-sulbactam monotherapy | 7 |
| Trimethoprim/sulfamethoxazole monotherapy | 7 |
| Ceftazidime monotherapy | 6 |
| Cefepime plus amikacin | 4 |
| Levofloxacin monotherapy | 4 |
| Ceftriaxone monotherapy | 2 |
| Fosfomycin plus amikacin | 2 |
| Cefepime monotherapy | 1 |
| Tigecycline monotherapy | 1 |
| Cefepime plus fosfomycin | 1 |
| Fosfomycin plus gentamicin | 1 |
| Fosfomycin monotherapy | 1 |
| Fosfomycin plus cefoperazone-sulbactam | 1 |
| Penicillin G | 1 |
| Definitive treatment (n = 311) | |
| Carbapenem-based regimens | **68** |
| Meropenem monotherapy | 26 |
| Imipenem monotherapy | 4 |
| Ertapenem monotherapy | 1 |
| Carbapenem plus other antibiotics | 37 |
| Meropenem plus amikacin | 12 |
| Meropenem plus fosfomycin | 11 |
| Meropenem plus tigecycline | 6 |
| Meropenem plus trimethoprim/sulfamethoxazole | 2 |
| Meropenem plus tigecycline plus trimethoprim/sulfamethoxazole | 1 |
| Meropenem plus ampicillin-sulbactam | 1 |
| Meropenem plus fosfomycin plus amikacin | 1 |
| Imipenem plus gentamicin | 1 |
| Imipenem plus amikacin | 1 |
| Meropenem plus tigecycline plus fosfomycin | 1 |
| Piperacillin-tazobactam-based regimens | **2** |
| Piperacillin-tazobactam monotherapy | 1 |
| Piperacillin-tazobactam plus other antibiotics | 1 |
| Piperacillin-tazobactam plus trimethoprim/sulfamethoxazole | 1 |
| Polymyxins-based regimens | **159** |
| Polymyxin monotherapy | 43 |
| Polymyxin plus other antibiotics | 116 |
| Polymyxin plus meropenem | 61 |
| Polymyxin plus tigecycline | 16 |
| Polymyxin plus fosfomycin | 10 |
| Polymyxin plus ampicillin-sulbactam | 5 |
| Polymyxin plus imipenem | 5 |
| Polymyxin plus tigecycline plus meropenem | 5 |
| Polymyxin plus trimethoprim/sulfamethoxazole | 3 |
| Polymyxin plus fosfomycin plus meropenem | 3 |
| Polymyxin plus trimethoprim/sulfamethoxazole plus fosfomycin | 2 |
| Polymyxin plus cefepime | 2 |
| Polymyxin plus cefoperazone-sulbactam | 1 |
| Polymyxin plus fosfomycin plus cefoperazone-sulbactam | 1 |
| Polymyxin plus ertapenem plus amikacin | 1 |
| Polymyxin plus ertapenem plus meropenem | 1 |
| Ceftazidime-avibactam-based regimens | **29** |
| Ceftazidime-avibactam monotherapy | 16 |
| Ceftazidime-avibactam plus other antibiotics | 13 |
| Ceftazidime-avibactam plus amikacin | 4 |
| Ceftazidime-avibactam plus fosfomycin | 2 |
| Ceftazidime-avibactam plus polymyxin plus fosfomycin | 2 |
| Ceftazidime-avibactam plus imipenem | 1 |
| Ceftazidime-avibactam plus amikacin plus fosfomycin | 1 |
| Ceftazidime-avibactam plus polymyxin | 1 |
| Ceftazidime-avibactam plus amikacin plus tigecycline | 1 |
| Ceftazidime-avibactam plus tigecycline | 1 |
| Other antibiotic regimens | **53** |
| Trimethoprim/sulfamethoxazole monotherapy | 14 |
| Fosfomycin monotherapy | 14 |
| Levofloxacin monotherapy | 10 |
| Amikacin monotherapy | 4 |
| Cefepime plus amikacin | 2 |
| Fosfomycin plus amikacin | 2 |
| Amoxicillin-clavulanic acid monotherapy | 1 |
| Ciprofloxacin monotherapy | 1 |
| Ceftazidime plus amikacin | 1 |
| Levofloxacin plus trimethoprim/sulfamethoxazole | 1 |
| Trimethoprim/sulfamethoxazole plus fosfomycin | 1 |
| Tigecycline plus fosfomycin | 1 |
| Penicillin G | 1 |

30 patients did not receive any definitive antibiotic therapy

**Table S4. Standardized mean differences of baseline covariates adjusted by SIPTW analysis**

|  | Standardized mean difference (SMD) | |
| --- | --- | --- |
| Variable | Unadjusted | Adjusted |
| Age | 0.118 | 0.004 |
| Gender, male | 0.034 | 0.018 |
| Severely restricted health status before hospital admission | 0.173 | 0.074 |
| Late-onset HA-BSI | 0.022 | 0.022 |
| HA-BSI with *Acinetobacter* spp. | 0.203 | 0.008 |
| Source of HA-BSI | 0.215 | 0.007 |
| Charlson comorbidity index | 0.002 | 0.008 |
| SOFA score | 0.293 | 0.036 |
| Carbapenem resistance | 0.437 | 0.004 |

**Abbreviations;** SIPTW, stabilized inverse probability of treatment weighting; HA-BSI, hospital-acquired bloodstream infections; SOFA, sequential organ failure assessment; SMD, standardised mean difference. Inverse probability weights were estimated and truncated at the 99th percentile to reduce the influence of extreme weights. The stabilized weights had a mean of 1.3 and variance of 0.7, with no evidence of extreme values. Covariate balance improved substantially after weighting, with all standardized mean differences below 0.1

**Table S5. Primary and secondary outcomes among patients treated with EAAT or LAAT for monobacterial HA-BSI caused by GNB**

| All patients | EAAT (within 24h), n (%) | LAAT (>24h), n (%) | Adjusted OR (95% CI)* |
| --- | --- | --- | --- |
| 14-day ACM | 40 (14.7) | 175 (42.9) | 0.37 (0.23-0.58) |
| 28-day ACM | 71 (26.1) | 202 (49.5) | 0.67 (0.45-0.98) |
| All patients | **EAAT (within 6h), n (%)** | **LAAT (>6h), n (%)** | **Adjusted OR (95% CI)*** |
| 14-day ACM | 22 (10.8) | 193 (40.5) | 0.26 (0.15-0.46) |
| 28-day ACM | 45 (22.2) | 228 (47.8) | 0.62 (0.40-0.97) |

Abbreviations: ACM, all-cause mortality; EAAT, early appropriate antimicrobial therapy; LAAT, late appropriate antimicrobial therapy; HA-BSI, hospital-acquired bloodstream infections; GNB, Gram-negative bacteria; n, number; OR, odds ratio; CI, confidence interval.

*Adjusted for baseline confounders using inverse probability treatment weighting (IPTW) with pre-specified covariates: age, gender, general health status before admission, early (within 7 days of hospital admission) vs late-onset BSI (beyond 7 days of hospital admission), source of BSI, type of pathogen, Charlson comorbidity index, carbapenem resistance status, and SOFA score

Center participated in study (n = 23)

Patients included (n = 764)

Center excluded (n = 1)

Patients excluded (n = 60)*

Patients excluded (n = 24)

- Non-consecutive enrolment (n = 14)
- Community-acquired (n = 5)
- Retrospective inclusion (n = 3)
- Missing consent (n = 2)

Center participated in study (n = 22)

Patients included (n = 680)

**Supplementary figure S1. Flowchart of patient inclusion**

*60 patients from one center were excluded because of poor quality of data

**References**

1. European Committee on Antimicrobial Susceptibility Testing Breakpoint tables for interpretation of MICs and zone diameters, Växjö: EUCAST; 2024. Version 14.0. Available from:<https://www.eucast.org/fileadmin/src/media/PDFs/EUCAST_files/Breakpoint_tables/v_14.0_Breakpoint_Tables.pdf> (accessed 26 May 2025).
2. Vincent JL, Moreno R, Takala J, Willatts S, De Mendonça A, Bruining H, et al. The SOFA (Sepsis-related Organ Failure Assessment) score to describe organ dysfunction/failure. On behalf of the Working Group on Sepsis-Related Problems of the European Society of Intensive Care Medicine. Intensive Care Med 1996;22:707-10. https://doi.org/10.1007/BF01709751.
3. Grissom CK, Brown SM, Kuttler KG, Boltax JP, Jones J, Jephson AR, et al. A modified sequential organ failure assessment score for critical care triage. Disaster Med Public Health Prep 2010;4:277-4. <https://doi.org/10.1001/dmp.2010.40>.

**Case report form**

A multicenter Observational study of Hospital-acquired Bacterial Bloodstream Infection

V 4.1

10/09/2020

**Inclusion/Exclusion**

Subject ID:

**Inclusion criteria**

- Age ≥ 18 years
- Hospital-acquired Bloodstream Infection (HA-BSI)

**Exclusion criteria**

- Previous inclusion in the study
- End-of-life care
- Pregnant patients
- Community-acquired BSI

**Demographic data**

- Gender
- Weight (kg)
- Height (m)

**Administrative data**

- Date of hospital admission (day/month/year)
- Date of ICU admission (day/month/year), if exist
- Origin of hospital admission
- Home
- Long-term care or Nursing health facility
- Another hospital
- Type of admission
- Medical
- Surgical
- ICU
- Normal ward
- Emergency department

**Blood culture data**

- Timing of the index blood culture sampling as date (day/month/year) and time (e.g., 15:50)
- Presumed source of HA-BSI
- Primary bacteremia (no source of BSI or no portal of entry)
- Central venous line associated
- Pneumonia
- Pleural (empyema)
- Tracheobronchitis
- Peritonitis
- Biliary source
- Other intra-abdominal infectious sources
- Urinary tract
- Necrotizing fasciitis
- Other skin and soft tissue infections
- Bone and joint infections
- Endocarditis
- Central nervous system infections
- Other, please describe ……

**Type of causative bacterial species and their antimicrobial susceptibility data**

Dependent on the species that is selected in the eCRF a susceptibility pattern checklist will pop-up.

Possibility to enter multiple pathogens. Tables for pathogen specific antibiogram provided

Table 1: Aerotolerant Gram-positive bacteria

Table 2: Aerotolerant Gram-negative bacteria

Table 3: Strick anaerob

If Coagulase Negative Staphylococcus (or other common contaminants) is selected: please confirm there have been at least 2 positive blood cultures with the same pathogen (species and susceptibility profile) or infected material with the same pathogen and strong clinical suspicion of the blood culture not being a contaminant.

**Disease severity scoring**

**Underlying comorbid conditions (tick all underlying comorbidities)**

Dementia

Hemiplegia

Cerebrovascular disease

Peripheral vascular disease

Previous Myocardial infarction

Heart failure (NYHA 3)

Heart failure (NYHA 4)

Diabetes without end organ damage

Diabetes with end organ damage

Chronic renal disease

Renal disease, requiring chronic dialysis

Connective tissue disease

Peptic ulcer disease (gastro-duodenal)

Liver disease, mild to moderate

Liver disease, severe

Immunosuppression

- Steroids > 20 mg/day for at least 4 weeks or recent high dose steroids
- Systemic anti-cancer chemotherapy within 6 months
- Solid organ transplant
- AIDS (not only HIV positive)
- Other causes of immunosuppression (e.g., primary immunodeficiency disorder and the use of other immunosuppressive medications), please describe ……

COPD/Moderate severity chronic obstructive pulmonary disease

COPD/Severe chronic obstructive pulmonary disease

Solid organ malignancy (active only) without metastasis

Solid organ malignancy with proven metastasis

- Head and Neck
- Lung
- Stomach
- Colorectal
- Other gastro-intestinal
- Breast
- Gynecological
- Prostate
- Other, please describe ……

Hematological malignancy (Leukemia or lymphoma)

- Acute lymphocytic leukemia
- Acute myeloid leukemia
- Chronic Lymphocytic Leukemia
- Chronic Myelogenous Leukemia
- Non-Hodgkin lymphoma
- Hodgkin lymphoma
- Acute lymphocytic leukemia
- Other, please describe ……

# **Previous Health status**

- Prior good health; no functional limitation
- Mild to moderate limitation of activity because of a chronic medical issue
- Chronic disease producing serious but not incapacitation restriction of activity
- Severe restriction of activity due to disease (e.g., bedridden or institutionalized)

**Severity scoring of illness**

Please enter worse values/conditions of +/- 24h of HABSI onset

Cardiac arrest in the 48 hours preceding or the 24 hours following BC sampling

Inotrope medications (please enter max doses +/- 24h of HABSI onset)

- Adrenaline (max dose)
- Noradrenaline (max dose)
- Dobutamine (max dose)
- Dopamine (max dose)
- Vasopressin (max dose)

Status of ventilation (please enter the worst status of ventilation +/- 24h of HABSI onset)

-Listed from worst to best ventilation status-

- Invasive mechanical ventilation
- CPAP or non-invasive mechanical ventilation
- High flow oxygen support
- Low flow oxygen
- No oxygen requirement

Renal replacement therapy

| Heart rate | (min) | (max) |
| --- | --- | --- |
| Systolic Blood Pressure | (min) | (max) |
| Mean Arterial Pressure | (min) | (max) |
| Respiratory Rate | (min) | (max) |
| Glasgow Coma Scale | / 15 * | GCS was assessed with ongoing sedation |

Temperature (min) (max)

SPO2 (Pa02 will be entered for ICU patients)

FiO2 % (please enter paired SPO2/FiO2 for the worse value of the 24h)

-Please enter paired PaO2/FiO2 for the worse value of the 24h for ICU patients)-

Creatinine (max)

Bilirubin (max)

Platelet count (min)

**Previous antibiotics and colonization**

Colonization or previous infection with pathogens listed below prior the index HA-BSI

MRSA (Staphylococcus aureus isolates resistant to methicillin)

VRE (of Enterococcus spp. isolates resistant to vancomycin)

Carbapenem resistant *Enterobacterales*

# **Previous antimicrobial therapy**

Did the patient receive any antimicrobials in the 30 days prior to the bloodstream infection (other than those started for the index HA-BSI episode)

If yes,

Name of the antimicrobial (dropdown list)

**Antimicrobial therapy for the index HA-BSI episode**

Name of the antimicrobial (dropdown list)

Date (day/month/year) and time (hh:mm) of the first dose

Date of the last dose (with the same format described above)

Route (IV, aerosolized, oral, IM)

Was a loading dose administered (If yes, the dose as gram/milligram/unit)

Reason for antibiotic prescription (one from dropdown list)

- Empirical therapy for sepsis
- Targeted therapy for blood stream infection based on positive blood culture
- Targeted therapy for blood stream infection based on antibiogram results
- De-escalation based on antibiogram results (study infection)
- Escalation based on antibiogram results (study infection)
- 2^nd^ antibiotic for combination therapy (study infection)
- Other, please specify……….

Reason for stopping the antibiotic (one from dropdown list)

- Patient cured
- Duration of treatment completed
- Change to a different antibiotic, escalation
- Change to a different antibiotic, de-escalation
- Stopping an antibiotic, de-escalation
- Allergic reaction to the antimicrobial
- Adverse event attributed to the antimicrobial
- Other, please specify……….

# **Source control**

Was source control required If yes:

*(please enter each intervention for source control, if more than one repeat the table)*

Date and time of the source control intervention (dd/mm/yyyy , hh:mm)

Type of source control intervention (*select one from dropdown*)

Surgical

- Surgical abdominal
- Surgical cardiac
- Surgical thoracic and mediastinal
- Surgical Vascular
- Surgical Skin
- Surgical other, please specify

Percutaneous

- Percutaneous abdominal
- Percutaneous renal (including stent)
- Percutaneous vascular
- Percutaneous thoracic (including chest drain)
- Percutaneous mediastinal
- Percutaneous other, please specify

Catheter removal

Other, please specify

Was source control effective

- Completely
- Incompletely
- No, it has been attempted but ineffective

**Status at day 14**

Severity of illness at day 14

Please enter worse values/conditions of +/- 24h of HABSI onset

Cardiac arrest in the 48 hours preceding or the 24 hours following BC sampling

Inotrope medications (please enter max doses +/- 24h of HABSI onset)

- Adrenaline (max dose)
- Noradrenaline (max dose)
- Dobutamine (max dose)
- Dopamine (max dose)
- Vasopressin (max dose)

Status of ventilation (please enter the worst status of ventilation +/- 24h of HABSI onset)

-Listed from worst to best ventilation status-

- Invasive mechanical ventilation
- CPAP or non-invasive mechanical ventilation
- High flow oxygen support
- Low flow oxygen
- No oxygen requirement

Renal replacement therapy

| Heart rate | (min) | (max) |
| --- | --- | --- |
| Systolic Blood Pressure | (min) | (max) |
| Mean Arterial Pressure | (min) | (max) |
| Respiratory Rate | (min) | (max) |
| Glasgow Coma Scale | / 15 * | GCS was assessed with ongoing sedation |

Temperature (min) (max)

SPO2 (Pa02 will be entered for ICU patients)

FiO2 % (please enter paired SPO2/FiO2 for the worse value of the 24h)

-Please enter paired PaO2/FiO2 for the worse value of the 24h for ICU patients)-

Creatinine (max)

Bilirubin (max)

Platelet count (min)

**Day 28 follow-up**

# **28-day status**

- Alive in the ICU
- Alive in the Hospital
- Death in the ICU
- Death in the Hospital
- Discharged from the Hospital
- Date of ICU discharge (day / month / year):
- Date of Hospital discharge (day / month / year):
- Date of death (day / month / year):
